# *TOMM40* ‘523 Genotype Distinguishes Patterns of Cognitive Improvement for Executive Function in *APOE* *ɛ*3 Homozygotes

**DOI:** 10.3233/JAD-230066

**Published:** 2023-10-10

**Authors:** Amber Watts, Stephen Haneline, Kathleen A. Welsh-Bohmer, Jingtao Wu, Robert Alexander, Russell H. Swerdlow, Daniel K. Burns, Ann M. Saunders

**Affiliations:** a University of Kansas, Alzheimer’s Disease Research Center, Fairway, KS, USA; b Zinfandel Pharmaceuticals, Research Triangle Park, Chapel Hill, NC, USA; c Duke University, Durham, NC, USA; d Takeda Development Center Americas, Cambridge, MA, USA

**Keywords:** Alzheimer’s disease, *APOE*, cognition, executive function, longitudinal, *TOMM40*

## Abstract

**Background:**

*TOMM40* ‘*523* has been associated with cognitive performance and risk for developing Alzheimer’s disease independent of the effect of *APOE* genotype. Few studies have considered the longitudinal effect of this genotype on change in cognition over time.

**Objective:**

Our objective was to evaluate the relationship between *TOMM40* genotype status and change in cognitive performance in the TOMMORROW study, which was designed to prospectively evaluate an algorithm that includes *TOMM40* ‘523 for genetic risk for conversion to mild cognitive impairment.

**Methods:**

We used latent growth curve models to estimate the effect of *TOMM40* allele carrier (short, very long) status on the intercept and slope of change in cognitive performance in four broad cognitive domains (attention, memory, executive function, and language) and a combined overall cognitive score over 30 months.

**Results:**

*TOMM40* very long allele carriers had significantly lower baseline performance for the combined overall cognitive function score (B = –0.088, *p* = 0.034) and for the executive function domain score (B = –0.143, *p* = 0.013). Slopes for *TOMM40* very long carriers had significantly greater increases over time for the executive function domain score only. In sensitivity analyses, the results for executive function were observed in participants who remained clinically stable, but not in those who progressed clinically over the study duration.

**Conclusions:**

Our results add to the growing body of evidence that *TOMM40,* in the absence of *APOE* *ɛ*4, may contribute to cognitive changes with aging and dementia and support the view that mitochondrial function is an important contributor to Alzheimer’s disease risk.

## INTRODUCTION

A growing body of evidence suggests that *TOMM40*, a gene located next to *APOE* on chromosome 19, may influence the risk of developing Alzheimer’s disease (AD) [1]. Variants of this gene are associated with cognitive performance and rate of cognitive decline [2, 3], brain volume in dementia-associated regions [4–6], and amyloid-β-induced cellular damage [7]. The close proximity of *TOMM40* and *APOE* genes makes it challenging to disassociate the independent effects of each, especially given the outsized effects of *APOE* *ɛ*4 compared to most single nucleotide polymorphisms. Despite this linkage disequilibrium, *TOMM40* likely operates via a unique mechanism compared to the underlying processes of risk conferred by the *APOE* *ɛ*4 allele. *TOMM40* encodes a protein critical to mitochondrial function and likely affects AD risk through effects on mitochondrial bioenergetic processes [8], whereas the mechanisms through which *APOE* confers risk may associate with lipid transport between cells, given the essential functions of lipids related to neuronal growth, maintenance, repair, and synaptic plasticity [9].

A variable length homopolymeric T variation has been identified in intron 6 of the TOMM40 gene, rs 10524523 (‘523) and is associated with earlier age of onset for late-onset AD. The alleles at this locus have been grouped into short (S) long (L) and very long (VL) depending on the T length [10]. However, understanding the molecular effect of this variant on AD etiology has been difficult given the high linkage disequilibrium in this region of chromosome 19. One way to disentangle these potentially confounded effects of TOMM40 ‘523 and *APOE* *ɛ*4 is to study the effects of *TOMM40* ‘523 among *APOE* *ɛ*3 homozygotes, removing the well-established effect of *ɛ*4 alleles on cognitive function. Two previous studies focused solely on *APOE* *ɛ*3 homozygotes reported that carriers of two short alleles for *TOMM40* ‘523 had lower baseline cognitive performance [11] and steeper rates of decline over longitudinal follow up [2]. Studies that did not restrict their samples to *APOE* *ɛ*3 homozygotes have nevertheless confirmed independent effects of *TOMM40* from *APOE* *ɛ*4 in cognitive performance and decline [12–14], cognitive test-retest effects [15] and timing of dementia onset [10].

Few studies have conducted *TOMM40* genotyping in large samples with well-characterized cognitive performance across multiple occasions [2, 11, 14, 15]. The goal of the current paper is to evaluate the relationship between *TOMM40* genotype status and change in cognitive performance across 30 months of the TOMMORROW study, which was designed to prospectively evaluate an algorithm that includes *TOMM40* ‘523 for genetic risk for conversion to mild cognitive impairment (MCI).

## METHODS

### Study design

**Fig. 1:**
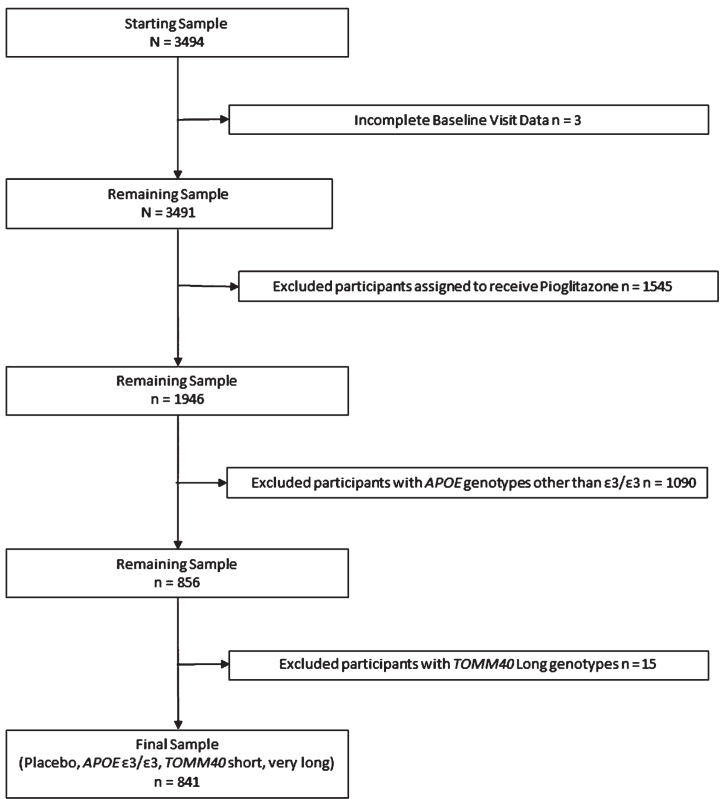
Inclusion and exclusion of participants from analysis.

The present study is a secondary analysis of data from the TOMMORROW study [16]. One of the primary objectives of the TOMMORROW study was to evaluate a genetic biomarker algorithm for timing of onset of MCI due to AD conferred by age, *APOE*, and *TOMM40* ‘*523*. The second primary objective was to test the efficacy of low dose pioglitazone to delay the onset of MCI due to AD in cognitively normal older adult participants. The study randomized participants considered to be at “high risk” to receive pioglitazone treatment or placebo and assigned those at “low risk” to placebo only. To evaluate changes in cognition without influence by experimental treatment, we excluded participants randomized to receive pioglitazone (see Fig. 1). Receiving the drug could potentially confound the effects of genotype combinations on cognitive change over time. Although the study stratified participants into high and low risk categories for conversion to MCI based on age and genotype, we did not use this stratification for the present analysis, instead combining all participants assigned to placebo into one sample comprising a more continuous distribution of risk. To address our research question regarding the effects of *TOMM40* ‘*523* independent of the effects of *APOE* *ɛ*4, analyses included only *APOE* *ɛ*3/*ɛ*3 homozygotes. Finally, we excluded participants who carried a *TOMM40* ‘*523* Long allele, the *TOMM40* allele typically linked to the *APOE* *ɛ*4 allele, to eliminate any confound of this association.

### Participants

A complete description of participant selection and procedures has been published elsewhere [16]. Briefly, the original study included participants aged 65–83 at the initial screening visit. Study enrollment occurred between 2013 and 2015 and included 57 clinical sites from the United States, United Kingdom, Australia, Switzerland, and Germany. To be included, participants had to be able to perform cognitive tests within normal range and be fluent in the language in which tests were administered. The study excluded participants who were determined to have a Clinical Dementia Rating (CDR) scale global score greater than 0 or objectively measured memory scores falling lower than 1.5 standard deviations below the normative mean. Other exclusion criteria were any diagnosis of cognitive impairment or dementia, significant psychiatric illness, alcohol or drug abuse, or other diseases contraindicated for use with pioglitazone. Due to the study’s unequal randomization by high and low risk for conversion to MCI, the most frequent reason for screening failure was being at low genetic risk for development of MCI.

### Procedures

The consent forms and process were approved by our institutional review board and was conducted in accord with the Helsinki Declaration of 1975. A three-visit enrollment process included a screening visit, a baseline visit, and a randomization visit. During the screening visit, participants were evaluated for cognitive status using the Mini-Mental State Examination (MMSE). If cognitively normal on this screen, blood samples were collected for genetic testing. At the baseline visit, inclusion and exclusion criteria for the study were reviewed and confirmed. The TOMMORROW neuropsychological test battery was administered to evaluate cognitive performance and confirm cognitive status as not impaired. If all entry criteria were met, participants were then randomized to active drug treatment or control based on a biomarker risk assessment algorithm. All low-risk participants were assigned to the placebo group and high-risk participants were randomized in a 1:1 ratio to drug or placebo.

Post-baseline neuropsychological assessments occurred every 6 months. The analysis described in this paper focused on the six-month post assessment follow up intervals, which we will refer to here as “waves.” The study duration was event-driven, where the primary endpoint event was conversion to MCI due to AD, and it anticipated a 5-year treatment phase to accumulate the predetermined event count. The study was terminated early following a pre-specified efficacy futility analysis, thus, available data for this analysis became sparse after 5 waves (30 months). To ensure accuracy and consistency across sites and time, the neuropsychological test results were centrally curated. To prevent practice effects common with repeated testing, the forms of the tests were counterbalanced across visits.

### Measures

#### Neuropsychological Test Battery

A team of experts designed the neuropsychological assessment battery to identify individuals exhibiting early stages of cognitive impairment. The study team selected instruments for the test battery known to be appropriate for use in preclinical stages of disease and that have known psychometric and normative data in English-speaking populations. The study team conducted validation studies to ensure consistency across multiple languages of assessment in study participants [17]. Each measure represented one of five broad domains of cognitive function associated with age-related declines and development of AD (memory, language, attention, executive function, visuospatial/praxis), with no fewer than two measures assessed per domain. We used alternative versions of verbal learning and visual memory tests to minimize practice effects. Composite scores were calculated for the memory, language, attention, and executive function domains based on the mean-derived z-scores (Baseline Mean/Baseline Standard Deviation) of selected tests in the domain using non-missing baseline values and adjusting for age and education levels. Composite scores were based on the average z-scores for tests in these four domains. For the present study, we omitted tests that were not part of the previously estimated composite scores due to ceiling effects in cognitively-normal participants (visuospatial function and naming). The z-scores for the Trail Making test part A and part B were adjusted (multiplied by – 1.0) so that all measures in the battery were scaled in the same direction with high scores indicating high/good performance and low scores indicating low/poor performance. Table 1 summarizes the domains and measures selected for composite calculations. As observed with other AD prevention studies, the trial cohort was healthier overall, had higher education levels, and had a high representation of Caucasians than the corresponding general older adult population.

**Table 1:** Neuropsychological Test Composite Scores by Cognitive Domain

Cognitive Domain	Tests
Attention	Wechsler Adult Intelligence Scale (WAIS-III) Digit Span Test – forward span
	Trail Making Test Part A
Episodic Memory	California Verbal Learning Test – 2nd Edition (CVLT-II)*
	Brief Visuospatial Memory Test – Revised (BVMT-R)*
Executive Function	Trail Making Test Part B
	Wechsler Adult Intelligence Scale (WAIS-III)
	Digit Span Test – backward span
Language	Semantic fluency (animals)
	Lexical/phonemic fluency (F, A, and S)

The scores for the tests in each domain were averaged to form a composite score, if there were no missing data. *Alternative versions of these tests were administered to minimize practice effects.

#### APOE and TOMM40 genotyping procedures

The TOMMORROW trial used Sanger sequencing methods followed by capillary electrophoresis to ascertain the *TOMM40* ‘523 poly-T length and a pyrosequencing assay to determine the *APOE* genotype. Genetic testing was performed under an FDA Investigational Device Exemption in a College of American Pathologists and Clinical Laboratory Improvement Amendments accredited environment. Poly-T lengths were used to classify each *TOMM40* allele according to the S, L, and VL convention from Roses et al. [10, 18].

#### Covariates

We included three covariates in the analyses age, sex, and years of education. Self-reported years of age and education are important predictors of cognitive performance. Self-reported sex was included as a potential predictor of cognitive performance, varying by cognitive domain. These covariates were collected by self-report at the screening visit. Educational attainment values were standardized using reported years of formal education and highest degree along with country-specific tables to assign years of education to report.

### Statistical analysis

We used latent growth curve models to estimate the association of *TOMM40 VL* carrier status with the intercept and slope of cognitive change over five waves adjusting for covariates (age, years of education, and sex). To estimate group differences between participants who remained clinically stable (i.e., CDR = 0) and those who progressed clinically (i.e., CDR > 0), we conducted multiple group growth curve models. We estimated change in R^2^ between models with and without the predictor variable (*TOMM40* allele status) as a measure of effect size, anticipating that effect sizes for single genes on cognition would be small to very small, on the order of 1% to 2%, as shown in prior similar research [11]. Missing data due to attrition was accounted for using a full information maximum likelihood algorithm with the understanding that data are likely not missing at random. To evaluate model fit, we used the ratio of *χ*^2^ to the model degrees of freedom and the Root Mean Squared Error of Approximation (RMSEA), a measure of the discrepancy between predicted and observed model values. Values closer to 0 indicate better fit (preferred values are < 0.09). Typically, these multiple fit indices are considered together as opposed to relying on any one indicator by itself. These latent models estimate all the pathways simultaneously which allows us to avoid multiple testing-induced inflation of type 1 error.

We estimated the statistical power for a latent growth curve model using Monte Carlo simulation following the guidelines of Muthén & Muthén [19]. The estimate included a normally distributed continuous dependent variable (i.e., cognitive scores), a categorical predictor (i.e., presence of TOMM40 very long alleles 0, 1), and adjustment for three covariates (i.e., age, sex, education). We estimated the power for five waves of longitudinal data based on the sample size of 841 observations available at baseline. The number of repetitions was set to 10,000 as recommended. We modeled missing data to match the pattern of longitudinal attrition observed in the data. The average was 7% attrition between waves [range 5% to 11%] with wave 5 representing 74% of the baseline observations. We estimated the effect size of the relationship between the independent and dependent variables to be small (i.e., β= 0.10 for the estimate of the slope of change over time reflecting an effect size of 0.32). Given a sample size of 841, we have 0.60 power to detect a small effect of *TOMM40 VL* (presence or absence) on the slope of change over time. All other estimates of independent variables on intercept and slope achieved an estimated power of 0.80 or greater. The small effect sizes of single genes on behavioral outcomes are well documented and require very large sample sizes to attain adequate statistical power. See Discussion for further detail.

## RESULTS

We report descriptive statistics in Table 2. There were no statistically significant differences in mean age or years of education between carriers and non-carriers of *TOMM40* VL alleles (*p* > 0.38). Using a *χ*^2^ test with Fisher’s exact estimation, there was no difference in the number of females and males in the groups with and without *TOMM40* VL alleles (*χ*^2^(df) = 2.62 (1), *p* = 0.113).

**Table 2:** Demographics

	Starting Sample	Remaining Sample			Final Sample		
	All	Low-risk placebo	High-risk placebo	All	Low-risk placebo	High-risk placebo	All
	*n* = 3,494	(*n* = 432)	(*n* = 1,514)	(*n* = 1,946)	(*n* = 276)	(*n* = 565)	(*n* = 841)
Age (years)*
Mean (SD)	74.0 (5.3)	70.3 (4.0)	74.6 (5.3)	73.7 (5.3)	70.1 (3.7)	78.8 (2.3)	76.0 (5.0)
Range	(65–83)	(65–83)	(65–83)	(65–83)	(65–81)	(68–83)	(65–83)
Age
<75 y	1650 (47.2%)	359 83.1%)	631 (41.7%)	990 (50.9%)	230 (83.3%)	4 (0.7%)	234 (27.8%)
≥75 y	1844 (52.8%)	73 (16.9%)	883 (58.3%)	956 (49.1%)	46 (16.7%)	561 (99.3%)	607 (72.2%)
Gender
Male	1573 (45.0%)	174 (40.3%)	666 (43.9%)	840 (43.1%)	115 (41.7%)	267 (47.35)	382 (45.4%)
Female	1921 (55.0%)	258 (59.8%)	848 (56.1%)	1106 (56.9%)	161 (58.3%)	298 (52.7%)	459 (54.6%)
Race
White	3368 (96.4%)	420 (97.2%)	1461 (96.5%)	1881 (96.7%)	271 (98.2%)	548 (97.0%)	819 (97.4%)
Black or African American	87 (2.5%)	10 (2.3%)	38 (2.5%)	48 (2.5%)	4 (1.4%)	9 (1.6%)	13 (1.5%)
Other	39 (1.1%)	2 (0.5%)	15 (1.0%)	17 (0.9%)	1 (0.4%)	8 (1.4%)	9 (1.1%)
Ethnicity
Non-Hispanic/ Latino Caucasian	3455 (98.9%)	424 (98.1%)	1495 (98.7%)	1919 (98.6%)	270 (97.8%)	554 (98.1%)	824 (98.0%)
Hispanic or Latino	39 (1.1%)	8 (1.9%)	19 (1.3%)	27 (1.4%)	6 (2.2%)	11 (1.9%)	17 (2.0%)
Years of formal education
Mean (SD)	14.7 (3.0)	14.7 (2.9)	14.7 (2.9)	14.7 (2.9)	14.6 (2.9)	14.7 (3.1)	14.7 (3.0)
Range	(2–20)	(2–20)	(3–20)	(2–20)	(7–20)	(6–20)	(6–20)
*Tomm40*
S/S	441 (12.6%)	101 (23.4%)	173 (11.55)	274 (14.1%)	87 (31.5%)	161 (28.5%)	248 (29.5%)
S/VL	1005 (28.8%)	183 (42.4%)	428 (28.4%)	611 (31.4%)	136 (49.3%)	404 (71.5%)	540 (64.2%)
VL/VL	182 (5.2%)	98 (22.7%)	37 (2.6%)	135 (6.9%)	53 (19.2%)		53 (6.3%)
S/L	539 (15.4%)	50 (11.6%)	235 (15.5%)	285 (14.6%)
L/VL	1160 (33.2%)		562 (37.1%)	562 (28.9%)
L/L	167 (4.8%)		79 (5.2%)	79 (4.1%)
Baseline MMSE
Mean (SD)	28.5 (1.4)	28.7 (1.3)	28.5 (1.4)	28.6 (1.4)	28.8 (1.3)	28.4 (1.3)	28.5 (1.4)
Range	(23–30)	(25–30)	(20–30)	(20–30)	(25–30)	(20–30)	(20–30)

**Fig. 2:**
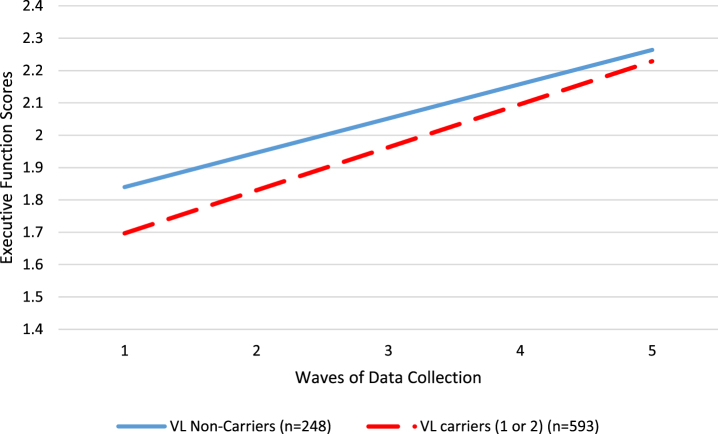
Differences in executive function performance between *TOMM40* VL allele carriers (red dashed line) and non-carriers (blue solid line).

We used latent growth curve models to evaluate the role of *TOMM40* VL carrier status (carriers (1 to 2 alleles) versus non-carriers (0 alleles)) on the intercept (baseline cognitive score) and slope of change in cognitive scores over five waves of data collection (in 6 month increments for a total of 2 years), adjusting for covariates age, sex, and years of education. Table 3 shows the results of the models. *TOMM40* VL carriers had significantly lower baseline performance for the overall cognitive function score (B = –0.088, *p* = 0.034; *Δ*R^2^ = 0.01) and for the executive function domain score (B = –0.143, *p* = 0.013; *Δ*R^2^ = 0.01). *TOMM40* VL carriers improved significantly more rapidly for only for the executive function domain score (B = 0.027, *p* = 0.030; *Δ*R^2^ = 0.03). See Fig. 2 for a visual illustration of the differences between *TOMM40* VL carriers and non-carriers in the executive function domain. Note that changes in performance overall were in the positive direction, so a positive slope indicates more rapidimprovement.

**Table 3:** Results of Latent Growth Curve Models, unstandardized estimates

	Overall Cognition B, p	Memory B, p	Attention B, p	Executive Function B, p	Language B, p
*Intercept*
Age	–0.028, < 0.001	–0.028, < 0.001	–0.028, < 0.001	–0.033, < 0.001	–0.025, < 0.001
Sex	0.107, 0.005	0.421, < 0.001	–0.092, 0.063	–0.001, 0.988	0.109, 0.049
Education	0.045, < 0.001	0.042, < 0.001	0.033, < 0.001	0.052, < 0.001	0.056, < 0.001
*TOMM40* VL (Carrier/Non-Carrier)	–0.088, 0.034	–0.061, 0.287	–0.086, 0.104	–0.143, 0.013	–0.065, 0.273
*Slope*
Age	–0.002, 0.001	–0.005, < 0.001	–0.001, 0.433	–0.002, 0.092	–0.002, 0.161
Sex	–0.008, 0.211	–0.023, 0.030	0.006, 0.619	–0.009, 0.447	–0.007, 0.522
Education	0.001, 0.569	–0.002, 0.328	0.000, 0.829	0.003, 0.102	0.000, 0.869
*TOMM40* VL (Carrier/Non-Carrier)	0.008, 0.233	–0.003, 0.787	0.016, 0.177	0.027, 0.030	–0.007, 0.572
*Model Fit*
*χ^2^ (df)*	27.72 (22)	83.03 (22)	12.41 (22)	28.66 (22)	12.28 (22)
*RMSEA*	0.018	0.057	0.000	0.019	0.000
*CFI*	0.99	0.98	1.00	0.99	1.00

To evaluate whether these relationships differed between clinically stable participants (i.e., CDR = 0 at all timepoints, *n* = 679) and those who progressed clinically (CDR > 0 at any point, *n* = 161), we repeated the models in a multiple group analysis. Results indicated that the relationship between *TOMM40* carrier status and executive function was present only in the clinically stable group (intercept B = –0.163, *p* = 0.006, slope B = 0.027, *p* = 0.004), but not in the group who progressed clinically (intercept B = –0.018, *p* = 0.904, slope B = 0.018, *p* = 0.551). The pattern of results in the other cognitive domains was consistent with the analysis of the total sample. For the full results of the sensitivity analysis, see Supplementary Table 1.

## DISCUSSION

Our results suggest that *TOMM40* VL allele carriers display a different pattern of cognitive performance and longitudinal change compared to those who do not carry a VL allele. In particular, *TOMM40* VL allele carriers started at lower levels at baseline in overall cognitive scores and in executive function domain scores but showed greater rates of improvement over time in executive function, nearly catching up with those who carried two *TOMM40* S alleles. It is worthwhile to note that, in sensitivity analysis, these results appeared to be driven by clinically stable participants, and not observed among the smaller subsample of participants who progressed clinically toward impairment. Previous studies report mixed results regarding which allele combination (short, long, very long) appears to be most advantageous in protecting cognitive performance and buffering against cognitive decline. Three prior longitudinal studies reported that VL carriers demonstrated superior performance at baseline in a variety of cognitive domains [3, 11, 14], though Yu et al. [2] reported no differences in baseline performance. Regarding change over time, three studies showed that VL carriers had a longitudinal advantage [2, 3, 20], while two showed that S carriers had slower declines [14] or stronger learning effects [15]. The findings of the current study suggested that the disadvantage seen for VL carriers at baseline, lessened over time by a faster rate of improvement, adding yet another pattern of results for consideration. Because our results showed this effect in those who remained unimpaired over the study duration, it is important to consider that *TOMM40* may have different effects in individuals with cognitiveimpairment.

Signs of antagonistic pleiotropy are common in research on the effects of genotypes on behavioral outcomes over time. That is, a genotype that appears to have an advantage in earlier stages of life may confer a disadvantage later in life. Some research has shown that *APOE* *ɛ*4 carriers outperform non *ɛ*4 carriers earlier in life, but decline more rapidly or show less cognitive resilience over time [21]. One conclusion we draw from these mixed findings in existing literature is that timing of assessment is critical for understanding who will appear to perform better on any cross-sectional occasion. Cross-sectional findings cannot substitute for longitudinal evaluation of cognitive change. It is also important to place our findings in the context of cognitively normal participants who are unlikely to decline over the span of two to three years, especially on cognitive assessments designed to detect early signs of dementia. Previous research has been mixed in its level of detailed characterization of the cognitive status of participants included in analyses. Patterns of cognitive decline observed among individuals at risk for developing dementia are likely to differ from patterns of improvement over time (likely due to practice effects and rates of learning) that are seen in studies of cognitively normal older adults, such as the sample included in the present study.

Previous studies provide clues about potential confounds that lead to mixed results across studies including age at time of cognitive testing [15], family history of AD [3], and *APOE* *ɛ*4 carrier status [2, 11], all of which may moderate these observed associations between *TOMM40* carrier status and cognitive performance and decline. Caselli et al. [15] reported a diminished test-retest effect for memory performance in *TOMM40* VL carriers only among the younger portion of their sample (younger than age 60). Willette et al. [3] highlighted that *TOMM40* VL carriers had better memory performance, but only when they were also negative for a family history of AD. Arpawong [12] demonstrated that the effects of *APOE* *ɛ*4 genotype are separable from the effects of *TOMM40* genotype, reporting that *APOE* was associated with delayed memory recall, while *TOMM40* was associated with immediate memory recall. It is reasonable that in the present study we observed genotype differences in executive function, as opposed to language, attention, or memory in high functioning, cognitively normal older adults, as executive function tasks are some of the earliest to decline as part of normal cognitive aging [22]. Of note, due to ceiling effects in our tests of visuospatial function, we were unable to draw conclusions about effects of *TOMM40* genotype on this domain of cognitive performance that has been observed in previous studies [23]. Previous studies of the effects of *TOMM40* genotype have reported influences on cognitive performance in a variety of domains including memory, visuospatial function, and language [2, 3, 11, 12, 14]. Our results suggest executive performance, which is typically associated with frontal lobe function, is particularly sensitive to *TOMM40* genotype. We are unaware of a strongly established biological mechanism, such as regional variations in brain TOMM40 expression, that might account for this finding. *TOMM40* has been found to associate with white matter integrity, though not restricted to the frontal lobe [24]. Advancing age is known to particularly affect executive function performance [25], as opposed to the amnestic state that presents as the most common AD clinical manifestation. Perhaps this indicates *TOMM40*’s effects on cognition pertain to the aging axis in cognitively normal older adults, rather than the more classical AD-associated bimesiotemporal axis. Since our findings contrast with previous literature, it is also possible that this may reflect limitations of measurement or study design rather than true underlying mechanisticrelationships.

Studies of single nucleotide polymorphisms as predictors of behavioral outcomes, such as cognitive performance, typically report very small effect sizes. This may be in part due to complex interactions of multiple genes that are not included in the analyses, and in part due to the difficulty in accurate measurement of behavioral traits compared to more objectively observed traits [26, 27]. The “fourth law of behavior genetics” suggests that complex behaviors are associated with many genes, each of which can only account for a tiny fraction of the total variability in the behavior [28]. Genes with easily detectable effect sizes such as *APOE* are rare[27].

Despite these disadvantages, there is firm justification for evaluating the role of *TOMM40* on cognition. A body of research consistently suggests it plays a role in cognition and there are well-described mechanisms through which *TOMM40* might influence AD risk and cognitive aging [8]. *TOMM40* encodes a critical mitochondrial protein and different ‘523 poly-T lengths very likely affect neural mitochondrial function. The effects of the different *TOMM40* ‘523 alleles on mitochondrial function itself are currently not well characterized; however, ‘523 poly-T length is known to influence *TOMM40* transcription, and ultimately, protein levels [18, 29].

We chose to use growth-curve modeling because it allows us to estimate the trajectories of change over time and use predictors such as *TOMM40* carrier status to describe group differences in the intercepts and slopes of the change in cognitive performance over time. We observed the effects of *TOMM40* VL alleles on change over time only in the domain of executive function in this sample of high functioning, cognitively normal older adults. These results suggest that carriers of one or more VL *TOMM40* allele may have advantages over time in the rates of cognitive improvement. Many studies of cognitive change over time in older adults display declines over years. By contrast, we did not detect declines, only greater or lesser degrees of improvement, likely due to the high functioning nature of our sample. These contrasting results suggest that participant age and timing of the arc of cognitive change during cognitive testing are important factors to account for when comparing findings across studies. Previous studies of the role of *TOMM40* on longitudinal cognitive change vary in their inclusion of participants with cognitive impairment or clinically confirmed normal cognition. It is unclear from these studies whether participants were high functioning overall or included a wider range of cognitive status including normal cognition, MCI, and AD. Some studies did not report whether the cognitive status of their participants remained normal over time.

The present study was undertaken using the TOMMORROW dataset as it included a rigorously administered and curated neuropsychological testing program [30], included a large number of subjects that would enable analysis without the confounding influence of the *APOE* *ɛ*4 allele, and the data on the genetic and cognitive evaluation was collected prospectively in a blinded fashion thereby reducing the possibility of bias. However, our study has several limitations. It is possible that our observation period was too short (total of 30 months) to detect age-related declines in the cognitive performance of pre-symptomatic older adults. This is shorter than the follow-up period of previous reports [2, 11, 14, 15]. Thus, our ability to detect cognitive change is limited, particularly in our high-functioning sample selected to maximize the distinction between high and low risk for conversion to MCI. Although the test battery was designed to detect early evidence of change, it may not be sufficiently sensitive in this cohort. As a clinical trial cohort, our sample is highly educated, in better health than the general population, and is mostly Caucasian. Furthermore, the clinical trial population reflects the randomization criteria utilized in the TOMMORROW study and therefore is not representative of the general older adult population. We excluded participants who were determined by CDR scale to be not cognitively normal or memory scores lower than 1.5 standard deviations below the mean, thus ensuring a high performing sample and limiting our generalizability. Finally, though the present study was unable to conduct neuroimaging to identify the neural mechanisms by which *TOMM40* may influence cognition, we refer readers to relevant studies of the effects of *TOMM40* on areas of the brain that are commonly associated with memory and visuospatial function [4, 6]. To our knowledge, no associations with areas of the brain associated with executive function (i.e., prefrontal cortex) have been reported. Finally, our study did not use biomarkers to evaluate the mechanisms by which *TOMM40* may influence brain or cognitive function. Many new AD biomarkers and imaging techniques have become widely available since to the initiation of this study and would not have been practical to implement in such a large, globally enrolledcohort.

Unique contributions of the present study include exclusion of *APOE *ɛ*4* alleles to facilitate decoupling of the *APOE *ɛ*4-TOMM40* ‘523 linkage disequilibrium, multiple cognitive tests summarized into distinct cognitive domains, and representation of participants from numerous countries around the world. Our results add to the growing body of evidence that *TOMM40,* in the absence of *APOE* *ɛ*4, may contribute to cognitive change with aging and dementia. Further, our findings support the increasingly accepted view that mitochondrial function is an important contributor to AD risk [31, 32]. These findings should be interpreted in the context of a growing body of converging evidence regarding the association of *TOMM40* ‘523 with age at dementia onset [10], changes in brain volumes in dementia specific regions [4–6], and cognitive performance and decline [2, 3]. *TOMM40* ‘523 merits investigation as a means of better understanding mechanisms, methods of early detection, and potential treatments for Alzheimer’s disease among those who do not carry an *APOE* *ɛ*4 allele.

## Supplementary Material

Supplementary Material

## Data Availability

The datasets, including the redacted study protocol, redacted statistical analysis plan, and individual participants data supporting the results reported in this article, will be made available within 3 months from initial request, to researchers who provide a methodologically sound proposal. The data will be provided after its de-identification, in compliance with applicable privacy laws, data protection and requirements for consent and anonymization. Research proposal applications can be made to www.Vivli.org where they will be reviewed and approved by an independent review panel. After a signed data access agreement has been received, the study’s protocol, statistical analysis plan, blank case report forms, clinical study report, de-identified individual patient data, and a data dictionary defining each field in the dataset will be made available at www.Vivli.org.
