# Supplementary material for: TOMM40 ‘523 Genotype Distinguishes Patterns of Cognitive Improvement for Executive Function in APOE ɛ3 Homozygotes
Source: J Alzheimers Dis. 2023 Oct 10;95(4):1697–707. doi: 10.3233/JAD-230066 (PMC10578241; doi:10.3233/JAD-230066)
Supplement: Supplementary Material [file jad-95-jad230066-s001.pdf]

Supplementary Material

TOMM40 '523 Genotype Distinguishes Patterns of Cognitive Improvement for Executive Function in *APOE* ε3 Homozygotes

Supplementary Table 1. Results of Multigroup Latent Growth Curve Models, unstandardized estimates

**CLINICALLY STABLE**<br>**n = 679**	Overall Cognition<br>B, p	Memory<br>B, p	Attention<br>B, p	Executive Function<br>B, p	Language<br>B, p
Intercept					
Age	-0.025, <0.001	-0.025, <0.001	-0.027, <0.001	-0.028, <0.001	-0.021, <0.001
Sex	0.105, 0.011	0.419, <0.001	-0.120, 0.027	-0.019, 0.139	0.136, 0.027
Education	0.042, <0.001	0.034, <0.001	0.027, 0.003	0.054, <0.001	0.053, <0.001
TOMM40 VL (Carrier/Non-Carrier)	-0.079, **0.074**	-0.030, 0.629	-0.109, 0.060	-0.163, **0.006**	-0.019, 0.768
Slope					
Age	-0.002, 0.007	-0.004, 0.001	-0.001, 0.338	-0.002, 0.118	-0.001, 0.327
Sex	-0.015, 0.036	-0.030, 0.007	0.009, 0.481	-0.019, 0.139	-0.018, 0.133
Education	0.000, 0.947	-0.003, 0.088	0.003, 0.166	0.001, 0.635	0.000, 0.813
TOMM40 VL (Carrier/Non-Carrier)	0.004, 0.639	-0.006, 0.647	0.011, 0.428	0.027, **0.044**	-0.020, 0.132
**CLINICALLY PROGRESSED**<br>**n = 161**	Overall Cognition<br>B, p	Memory<br>B, p	Attention<br>B, p	Executive Function<br>B, p	Language<br>B, p
Intercept					
Age	-0.033, 0.001	-0.029, 0.022	-0.023, 0.086	-0.040, 0.011	-0.039, 0.003
Sex	0.030, 0.744	0.295, 0.011	-0.019, 0.873	-0.044, 0.757	-0.065, 0.588
Education	0.052, <0.001	0.065, 0.001	0.054, 0.006	0.034, 0.136	0.062, 0.002
TOMM40 VL (Carrier/Non-Carrier)	-0.098, 0.317	-0.147, 0.242	0.021, 0.873	-0.018, **0.904**	-0.244, 0.060
Slope					
Age	-0.003, 0.099	-0.009, 0.003	0.001, 0.669	-0.002, 0.092	-0.002, 0.455
Sex	0.008, 0.630	-0.015, 0.601	-0.006, 0.824	-0.026, 0.353	0.025, 0.326
Education	0.002, 0.463	0.002, 0.731	-0.008, 0.045	0.011, 0.015	0.003, 0.481
TOMM40 VL (Carrier/Non-Carrier)	0.024, 0.150	-0.001, 0.964	0.035, 0.208	0.018, **0.551**	0.038, 0.167
Model Fit					
χ^2 *(df)*	43.71 (44)	116.48 (44)	44.15 (44)	50.57 (44)	37.74 (44)
RMSEA	0.000	0.063	0.003	0.019	0.000
CFI	1.00	0.98	1.00	0.998	1.00
